# Bioengineered microfluidic blood-brain barrier models in oncology research

**DOI:** 10.1016/j.tranon.2021.101087

**Published:** 2021-04-14

**Authors:** Robin Augustine, Ahmad H. Aqel, Sumama Nuthana Kalva, K.S. Joshy, Ajisha Nayeem, Anwarul Hasan

**Affiliations:** a Department of Mechanical and Industrial Engineering, College of Engineering, Qatar University, 2713 Doha, Qatar; b Biomedical Research Center (BRC), Qatar University, PO Box 2713 Doha, Qatar; c Department of Biotechnology, St. Mary's College, Thrissur 680020, Kerala, India

**Keywords:** Blood-brain barrier, BBB models, Microfluidics, Microenvironment, Cancer, Metastasis

## Abstract

Metastasis is the major reason for most brain tumors with up to a 50% chance of occurrence in patients with other types of malignancies. Brain metastasis occurs if cancer cells succeed to cross the ‘blood-brain barrier’ (BBB). Moreover, changes in the structure and function of BBB can lead to the onset and progression of diseases including neurological disorders and brain-metastases. Generating BBB models with structural and functional features of intact BBB is highly important to better understand the molecular mechanism of such ailments and finding novel therapeutic agents targeting them. Hence, researchers are developing novel *in vitro* BBB platforms that can recapitulate the structural and functional characteristics of BBB. Brain endothelial cells-based *in vitro* BBB models have thus been developed to investigate the mechanism of brain metastasis through BBB and facilitate the testing of brain targeted anticancer drugs. Bioengineered constructs integrated with microfluidic platforms are vital tools for recapitulating the features of BBB *in vitro* closely as possible. In this review, we outline the fundamentals of BBB biology, recent developments in the microfluidic BBB platforms, and provide a concise discussion of diverse types of bioengineered BBB models with an emphasis on the application of them in brain metastasis and cancer research in general. We also provide insights into the challenges and prospects of the current bioengineered microfluidic platforms in cancer research.

## Highlights

• Discusses the structure and physiology of BBB.
• Various currently available BBB models.
• Development and characterization of bioengineered microfluidic BBB models.
• Application of bioengineered microfluidic BBB models in cancer research.
• Challenges and prospects of bioengineered microfluidic BBB models in the context of cancer research.

## Graphical abstract

**Figure:**
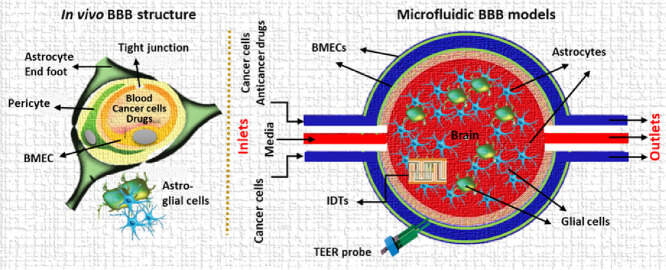

## 1 Introduction

Patients suffering from lung, breast, and colon cancer are highly susceptible to brain metastases [1]. 50% of lung cancer patients and 30% of breast cancer patients develop metastasis with a significant amount of them occurring in the brain [2,3]. It is approximated that brain metastasis happens in 20-40% of all types of cancers and causes intracranial tumors [4]. The high barrier property of the blood-brain-barrier (BBB) restricts even highly effective therapeutic agents (in other parts of body) from reaching brain and leads to ineffective therapeutic outcome [5], [6], [7]. Both *in vitro* and *in vivo* models have played a major role in the screening of suitable drug candidates for intracranial treatment. *In vivo* models which are mainly based on rodent models or other small animals provide a reliable platform for testing the BBB permeability of potential or approved drug candidates [8]. However, *in vivo* models are expensive and are applicable for low throughput screening assays at the final stages of drug testing [9]. Therefore, high throughput screening assays are necessary to perform the preliminary screening of a large number of potential drug candidates formulated by pharmaceutical companies [10]. In the context of brain tumors, this will ensure that promising drugs can be further tested while the ineffective ones can be modified or eliminated. *In vitro* models also allow a better understanding of the drug-cell interactions (for instance, drug-microvascular cell interactions), which is a major factor that determines the BBB permeability of drugs [11], [12], [13], [14]. I*n vitro* BBB models composed of BBB associated cells and extracellular matrix (ECM) mimetic hydrogels are promising candidates for studying the permeability of anticancer agents through BBB [14,15].

Studies indicated that unlike normal cells, cancer cells can cross BBB and result in metastatic brain tumors [16,17]. Thus, understanding the biological mechanisms behind cancer metastasis through BBB is highly important for developing new therapeutics which is a major challenge in developing new therapies against metastatic brain tumor [18]. Although studying the BBB function, its integrity, cancer metastasis through BBB, and the drug permeability through BBB are critical in oncology research, there is a general lack of validated standard models for investigating and unraveling such aspects [19], [20], [21]. It is also very important to develop BBB constructs that can recapitulate the composition of intact BBB to investigate the physiology of BBB [22,23].

An ideal model should maintain the identical BBB associated cell types along with their spatial distribution as closely as possible with that of *in vivo* structure [24]. Such models should possess many characteristic features including, disease-specific enzyme expression, functional expression of receptors and transporters, facilitate the paracellular transport of materials and, ease of permeability towards selective substances, and have high trans-endothelial electrical resistance (TEER) [25,26]. Such a system should also come up with advanced and highly accurate biosensors to detect and quantify drugs, biomolecules, and track cellular events. By using the principles of the promising field of bioengineering and its available tools, functional and economically viable engineered BBB models can be generated [27]. The engineered microfluidic BBB (µBBB) platforms will help to understand the mechanisms of cancer metastasis through BBB and support the successful development and application of new generation antitumor therapeutics aimed at prominent metastatic pathways [28]. *In vivo* mimicking, versatile, and commercial µBBB models can be fabricated by merging bioengineering, microfluidics, and biosensor technologies [29]. Many researchers utilized such combinations in recent years to develop µBBB platforms and attained excellent experimental outcomes [30]. This review provides a concise overview on the scientific and operational aspects of design, fabrication, testing, and application of bioengineered µBBB platforms, which will help to select a suitable model by understanding the constraints and potential possibilities in the context of oncology.

## 2 Biology of BBB

**Fig. 1:**
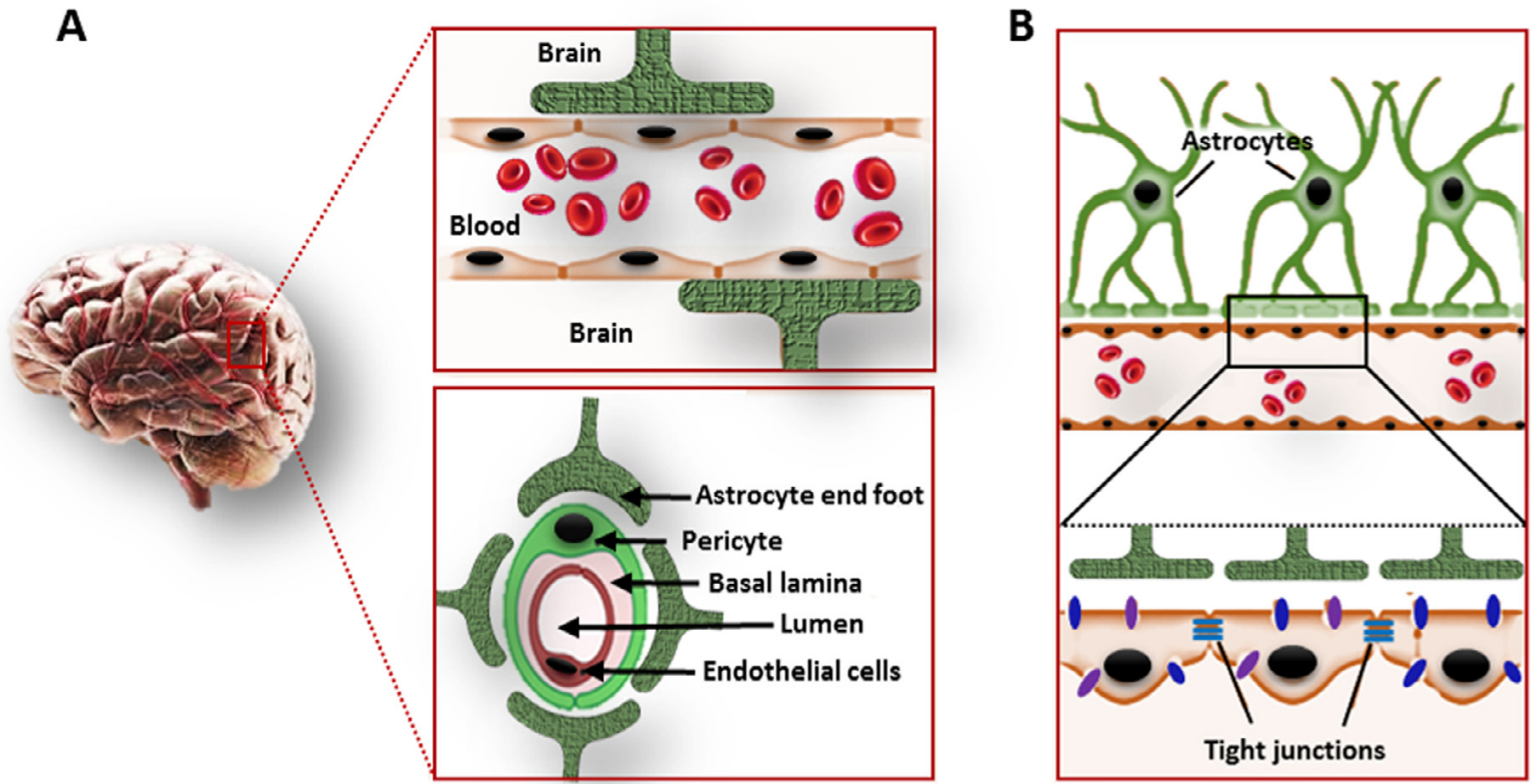
Scheme showing the cellular components of the blood-brain barrier (BBB). A. The innermost layer of BBB is formed by the closely packed arrangement of brain microvascular endothelial cells (BMECs). Then, the endothelial layer is surrounded by a basal lamina which is further surrounded by pericytes. Astrocytic end-foot constitutes the neurovascular unit which is in direct contact with neuronal tissue. B. A closely packed assembly of endothelial cells with the help of tight junction proteins forms the tight junctions in BBB.

The BBB performs a vital role in preserving central nervous system (CNS) homeostasis, keeps away potentially dangerous agents from CNS and restricts the CNS-entry of pathogenic organisms present in circulating blood [31]. In addition, it restricts the uptake of a major portion of all the probable neurotherapeutics in the blood-brain interface [32] and limits their therapeutic efficacy [33]. Fig. 1 depicts the cellular components and their organization in BBB. The BBB is mainly comprised of brain microvascular endothelial cells (BMECs) which are supported by a discontinuous layer of perivascular elements like pericytes, astrocytes, microglia, neurons, and perivascular macrophages [34,35]. The functioning of BBB is closely dependent on the interactions among nearby BMECs [36], [37], [38], [39], [40].To maintain the BBB integrity, endothelial cells form a closely packed monolayer that express a wide range of cell-cell adhesion proteins forming tight junctions (occluding junctions) [41]. This tight packing of cells effectively hinders the paracellular passage of compounds leaving the transcellular route as the main mechanism of transport across BBB [42,43]. However, multiple catalyzed transport systems exist in BBB that can bring molecules such as organic cations, amino acids, monocarboxylic acids, nucleosides, peptides, and nanoparticles into the brain (receptor-mediated transcytosis and carrier-mediated transport) and out of the brain [22,44]. The key efflux transporters that bring compounds into the blood and out of the brain include, breast cancer resistance protein (BCRP), P-glycoprotein (P-gp), glucose transporter (GLUT), BBB choline transport (BBB-ChT), and brain multidrug-resistance protein [45,46]. The BBB permits the entry of small ions like K^+^ and Cl^−^ through ion channels, permits the passage of ethanol and nicotine-like small lipophilic molecules, and allows the passage of polar molecules like glucose, lactate, and pyruvate through carrier-mediated transport. But, the transport of macromolecules like albumin, transferrin, insulin, leptin, and tumor necrosis factor-alpha (TNFα) across BBB involves adsorption-mediated transcytosis, receptor-mediated transport, and active efflux transport [47]. Luminal (facing capillary blood) and abluminal (facing brain interstitial fluid) endothelial cell membranes are involved in the passage of small lipid-soluble (lipophilic) agents, gaseous molecules like O_2_ and CO_2_, and also some of the CNS targeted drugs [48]. Several cargo-specific carrier proteins present in the endothelial membranes carry essential nutrients and compounds of high lipophilicity into the brain [45]. For example, phenylalanine resembling anticancer agent melphalan can be delivered across BBB by the Large neutral amino acid transporter 1 (LAT1) transporter [49].

## 3 Currently available BBB models

**Fig. 2:**
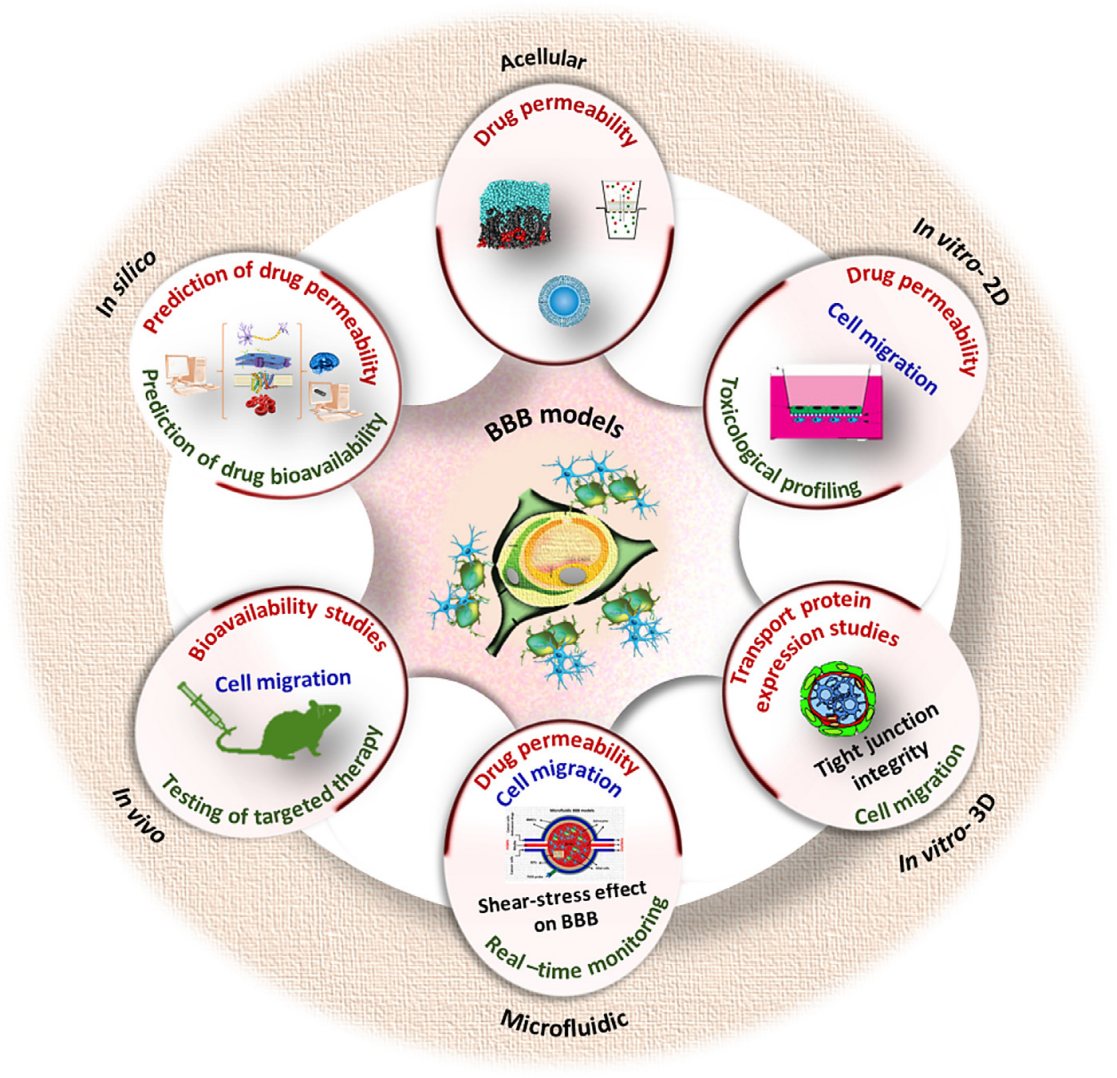
A scheme showing the application of various BBB models in drug development and pharmaceutical research.

Several *in silico, in vivo,* and *in vitro* approaches have been used to model BBB to study BBB function, molecular transport, drug permeability and pathophysiological events in BBB-brain interface [50]. A brief outline of such BBB models and their major applications in drug development and pharmaceutical research is provided in Fig. 2. Table 1 provides detailed information regarding the advantages and limitations of various BBB models.

**Table 1:** Advantages and limitations of various *in silico, in vivo, in vitro* or microfluidics models of BBB in the context of anticancer drug development.

Model types	Advantages	Limitations	Reference
*In silico*	• Low cost. • Suitable for high throughput preliminary drug screening studies.	• Applicable for calculating basic permeability based on drug chemistry only. • Not suitable for testing drug efficacy. • Must be further verified by *in vitro* and *in vivo* experiments.	[51], [52], [53]
*In vivo*	• Can closely mimic the complexity of human body. • Maintain natural microenvironment. • Generate large amount of reliable data.	• More than 80% of results obtained from in vivo models fail in clinical trials. • Animal-to-animal variability. • Expensive to conduct due to labor and animal costs. • High doses of drugs used in vivo are not suitable in high throughput screening.	[42,54,55]
Acellular *in vitro*{Using Immobilized Artificial Membrane assays (IAM), solid-supported lipid membrane assays (TRANSIL), or Parallel Artificial Membrane Permeability assays (PAMPA)}	• Fast, versatile, and low-cost. • Good prediction of BBB penetration for CNS classes of drugs.	• Not suitable for oral formulations based on low-solubility compounds.	[56], [57], [58], [59], [60], [61]
*In vitro* -2D models(Petri dishes, isolated brain microvessels, transwell systems)	• Simple, reliable and reproducible. • Highly convenient for high-throughput screening. • Used in studying signaling pathways, transporter kinetics, binding affinity calculation. • Cost effective. • High performance, long-term culture, easy to interpret.	• Incapable of explaining material transport or cancer cell migration across BBB. • Uncontrolled supply of oxygen, nutrients and biomolecules. • Cannot mimic the natural architecture of the stromal tissue or tumor tissue. • Lack of cell-cell and cell-extracellular matrix interactions. • No shear stress.	[62]
In vitro 3D models(ECM based models, Spheroid models, Dynamic *in vitro* (DIV) models	• Physiological cell to cell contacts relatively like in vivo conditions. • Cell-cell and cell-extracellular matrix interactions. • Can effectively be used to investigate drug delivery to the brain, brain vascular diseases, and mechanisms of metastasis. • Resistant to anticancer drugs, more accurate in assessing drug efficacy than 2D models.	• Expensive, time-consuming, fewer commercially available platforms. Labor intensiveness. • No consideration of shear stress (except DIV models)	[63]
Microfluidic *in vitro*	• Can simulate physiological fluid flow and shear stress. • Parameters can be controlled precisely by a computer. • Closely resembles the actual *in vivo* brain anatomy. • Ideal for studying cancer metastasis.	• Expensive. • Difficult to set up. • Highly skilled and well trained users are needed. • Lack of standardized quantification of parameters. • Difficult to visualize intraluminal compartment.	[20,64,65]

### 3.1 *In silico* models

In general, *in silico* models are based on the mathematical calculations on physicochemical parameters of BBB. Computer based models are also implemented in BBB research where the starting point is computer-assisted drug design [66]. With the aid of computer models, well-defined structure-activity interactions for the BBB permeation can be obtained and based on this data, the permeability of the drug can be predicted. To achieve this, physical and chemical properties such as surface area, van der Waals volume, hydrogen donor-acceptor characteristics, lipophilicity are considered [67].

The performance of *in silico* platforms depends heavily on the accessibility and reliability of existing experimental data. The most popular endpoint used in quantitative models is logBB, which presents the most readily obtainable experimental information [68]. Limited availability, relatively large variability and the complex nature of brain-penetration data imposes a considerable challenge for the development of predictive *in silico* BBB models. Computational models of BBB permeation involve simple rule-based, more sophisticated classification strategies, as well as quantitative models [69]. Current models utilize almost all model development techniques, from classical regression analyses to machine learning algorithms that have become increasingly popular in ADME (absorption, distribution, metabolism, and excretion) predictions. *In silico* BBB models use a wide variety of descriptors. As these models often consider logBB as the measure of BBB permeability, most of such methods are limited to membrane permeability and does not consider most of the biological factors [70]. Most of the available BBB permeation *in silico* methods relies on passive diffusion phenomena [71]. Permeation across the BBB is also persuaded by active transport systems in which P-gp is an important obstacle for the permeation of hydrophobic drugs. Hence it would be necessary to utilize a suitable prediction model with the capability to include the active transport phenomena [72]. Moreover, there is a critical need for approaches that merge different models considering both reflexive diffusion and dynamic drug delivery. *In silico* approaches have been successfully applied for the determination of drug permeability based on the structural information but are not able to provide much evidence on the efficiency of the drug in terms of BBB permeability [73]. The results obtained from computer models must be confirmed by *in vitro* and *in vivo* studies, as they cannot predict all the phenomena occurring in the biological system.

### 3.2 *In vivo* models

Various animal models have been developed in the past to study BBB function, to test the efficacy of brain targeted drugs, and for studying cerebral disorders. *In vivo* models of BBB such as xenograft human-mouse, genetically engineered mice, and pet animals with naturally occurring BBB abnormalities were used to develop BBB models. Some of the specific models include internal carotid artery perfusion [74], intracerebroventricular injection [75], microdialysis [76], intravenous injection [77], and quantitative autoradiography with brain imaging [78]. Establishment of Brain Uptake Index (BUI) and Brain Efflux Index (BEI) have remarkably increased our understanding of BBB uptake and efflux [79]. *In vivo* models generally provide reliable data for BBB drug permeability, as opposed to some newer cell-based *in vitro* technologies that focus more on mechanistic insights. This led to the development of several knock-out mice models to investigate the significance of apolipoprotein [80], angiotensinogen [81], tumor necrosis factor (TNF) [82], nitric oxide synthase 1 (NOS-1) [83], and P-gp [84] on BBB integrity and drug permeability.

The major benefit of *in vivo* models is that they can closely mimic the complexity of human physiology that cannot be fully achieved in *in-vitro* models [85]. On the contrary, labor cost and the use of large number of animals in various stages of the experiments make it more expensive to perform. Another major drawbacks of *in vivo* models is the sudden death of animals before completing the experiment due to the high dose of drugs that applied during experiments [86].

### 3.3 Acellular *in vitro* models

Acellular *in vitro* models typically estimate membrane permeability or membrane affinity using immobilized artificial membrane assays (IAM), solid-supported lipid membrane assays (TRANSIL), or parallel artificial membrane permeability assays (PAMPA). Salminen *et al* investigated several drugs using IAM chromatography and correlated the chromatographic capacity factor (kIAM) with *in vivo* logBB [87]. The IAM.PC.DD2 system adequately mimicked the BBB permeability phenomenon; however, data from both the IAM and MSC18 systems did not correlate with *in vivo* blood-brain distribution and brain-blood partition. The failure of IAM methods in modeling brain distribution is expected, as these methods are limited to equilibrium partitioning into liposomes rather than diffusion [88].

The PAMPA methodology developed by Kansy *et al* utilized egg lecithin in dodecane as the artificial membrane for predicting intestinal permeability [89]. Although several other phospholipid compositions have been investigated, Di and coworkers replaced egg lecithin in dodecane with porcine brain lipids in their modified PAMPA methodology [56]. One of the most important differences between artificial and natural membranes is that the former does not contain proteins (transporters and carriers) necessary for active transport. Therefore, artificial membranes can only estimate the BBB penetration of compounds by passive diffusion. Although PAMPA approach uses artificial membranes that lack BBB transporters, data obtained from this model correlated well with the Madin-Darby canine kidney (MDCK) model and *in vivo* rate measurements [90,91]. Carrara *et al* improved the BBB PAMPA methodology by further optimizing the monolayer constitution; porcine brain lipid in dodecane: hexane (1:1) gave the best predictive power compared with the cell-based MDCK-MDR1 model [92].

### 3.4 *In vitro* models

*In vitro* assays are crucial in cancer research as they can provide a controlled microenvironment which is necessary for obtaining consistent quantitative data [93]. These are considered as very important in BBB research because it is simple, more reliable, reproducible, and appropriate for high-throughput screening [94]. Various *in vitro* BBB models such as fiber-based dynamic *in vitro* BBB (DIV-BBB) platforms, 2D or 3D cell culture models, brain slice models, and microfluidic platforms have been introduced and used in cancer research [95]. Based on the occurrence of shear stress generated by the flow of blood, *in vitro* BBB models are divided into two categories: static and dynamic models. Static *in vitro* models do not recapitulate shear stress which is generated in *in vivo* models and are further categorized into monolayer and co-culture models according to the experimental setup. The monolayer models comprise only one major cell type (BMECs) and cannot recapitulate the interaction with other cell types. Because of its intrinsic oversimplified nature, this model is not suitable for the study of BBB integrity. However, it has been widely used in studying signaling pathways, transporter kinetics, binding affinity calculations, and in high throughput screening [96]. In the co-culture model, different cell lines are cultured in different places of the membrane. The luminal layer is formed by the endothelial cells cultured on the upper side and the cells co-cultured with astrocytes and/or pericytes in the bottom form the abluminal layer [97]. Although such systems are useful in understanding the structure and pathophysiology of tight junctions, their integrity, and the effect of various agents on them, they are incapable of explaining material transport or cancer cell migration across BBB. Moreover, growing information regarding the function of the microenvironment in the progression of cancer indicates that the crosstalk between cancer cells and host cells in a spatiotemporal manner can play an important function in metastatic cascade which is difficult to recapitulate in 2D *in vitro* systems [98]. Consequently, due to the lack of geometrical and microenvironmental features of the 2D *in vitro* models, it is crucial to develop 3D models that can mimic relatively similar *in vivo* environment.

In the past, transwell systems (a two-compartment cell culture well insert) were used as promising models, where multiple cell types were seeded over a porous membrane facilitating molecular transport or cell migration across the porous membrane to the lower compartment [99]. Also, earlier studies reported the applicability of transwell culture systems as a model to investigate the migration of breast cancer cell lines into the brain by crossing BBB [94]. *In vitro* 3D models of the BBB could enable precise control of the physiology, pharmacology, and pathophysiology of the BBB. They represent suitable tools to effectively investigate brain vascular diseases, drug delivery to the brain, and mechanisms of cell migration across the endothelial barrier with low cost and high throughput [72]. While conventional *in vitro* models fail to capture many of the *in vivo* features such as physiology and cellular microenvironment, the cell-laden 3D hydrogel containing microfluidic BBB model is a promising approach for creating bio-mimicking BBB models [94]. For studying cancer cell metastasis, hydrogel models have been usually employed [100]. In organotypic constructs, hippocampal thin sections are prepared and cultured on a supporting membrane surface in which cell types in brain slices are maintained to study the BBB features [101]. Measurement of trans-endothelial electrical resistance (TEER) and biomarker-based fluorescent immunostaining is convenient to be carried out in slice models [94].

Shear forces exerted on endothelial cells due to fluid flow is a major driving force that direct tight junction formation and appropriate endothelial polarization [102]. The absence of dynamic flow affects the rigidity of the barrier. In *in vivo* model, the TEER value is 1500–8000 Ω•cm^2^, but much lower resistance across the BBB is observed in static models, where the value is only 150–200 Ω•cm^2^ [26,103]. To overcome several issues related to *in vitro* static models and *in vivo* models, dynamic microfluidic chip-based BBB models have been proposed and developed in recent years. We will be detailing these highly advanced *in vitro* models in subsequent sections.

## 4 Microfluidic *in vitro* BBB platforms

Attempts have been made by the bioengineers to either mimic biological systems or alter them so that they can help to overcome several critical healthcare and clinical challenges. As an example, microfluidic chips developed with the support of microfabrication technologies allow the pumping of liquid reagents into micro-channels and performing biological, and chemical analysis cost-effectively and rapidly [104], [105], [106], [107], [108]. Major features of some of the reported µBBB models are given in Table 2. A relatively realistic model of the BBB can be generated by the use of microfluidic devices that mimic *in vivo* biological microenvironments using micro-electro-mechanical systems (MEMS) that have attained much attention recently [94]. An *in vitro* BBB-on-chip model is composed of: 1) a hydrogel component with several layers of ECs, pericytes, astrocytes, and neurons, 2) cell-laden hydrogels with the fluid–flow, shear stress, and controlled osmotic pressure with a vascular chamber for the supply of blood or medium, 3) a brain tissue chamber for the collection of molecules coming from blood vessel mimetic channels across BBB to brain component or vice versa and 4) an array of biosensors for the long-term monitoring of the BBB microenvironment. Owing to the importance of immune cells in BBB function and pathophysiology [109], BBB platforms that help to study the interaction of human T cells with component cells of BBB are also developed [110]. To generate an ideal µBBB-on-a-chip model, advances in microfluidics-based rational designing of BBB platforms and tissue engineering of 3D vascular systems are very necessary. Micro-scale engineering technologies are required for microfluidic chips to make channels, chambers, and valves using materials like silicon, glass, and quartz [111]. Sub-micrometer sized mechanical channels are formed with some macromolecular polymeric material. They have more resemblance to the intricate native brain microvasculature and its physical and biological microenvironments than the traditional *in vitro* BBB platforms [112].

**Table 2:** Various types of microfluidic BBB models, their advantages, disadvantages and applications.

Model	Advantages	Disadvantages	Application	Reference
BMEC-Derived Model	• Strong barrier integrity. • High TEER values.	• High fluid to cell ratio limits BBB mimicry.	• Drug permeability screening.	[21]
Dual-Chamber Membrane Model	• Easily replicable. • Allows for segregated manipulation of either side of the chamber membrane.	• Hydrophobic molecule adsorption. • Underwhelming TEER measurements.	• Studying drug toxicity and permeability.	[53]
SynVivo chip based Brain Model	• Readymade. • Flexibility to modify for different applications. • Can capture the true variability in permeability across cellular monolayer. • Used human cerebral microvascular endothelial cells and primary human astrocytes.	• Limited possibility to use for drug permeability studies.	• Useful for studying BBB-brain interactions at cellular and molecular level.	[124]
PDMS-Based Microfluidic Devices	• Increased BBB integrity when co-cultured with astrocytes. • Astrocyte- endothelial cells interaction was noticed.	• Susceptible to shrinkage-related problems, ex: air bubbles in channels.	• Drug permeability. • Drug screening.	[126,127,128]
Multichannel with integrated impedance sensors	• Possibility of label-free real time impedance measurement. • ZO-1 and GFAP expression. • Fibronectin/Matrigel coated.	-	• Drug screening. • Drug permeability studies. • Drug toxicity studies.	[102]
Three microchannel integrated system	• Polycarbonate membrane and Polyethylene terephthalate membrane based platform. • Mouse embryonic stem cells based cortical spheroids were used.	• No option for the direct TEER measurements. • Absence of astrocytes and pericytes.	• Suitable to study the neuroinflammation.	[129]
Hydrogel based vasculogenesis BBB model	• HUVEC and astrocytes were used. • CD31, ZO-1, and GFAP expression tested.	• Used umbilical cord endothelial cells instead of brain microvascular endothelial cells.	• Screening of brain targeted pharmaceuticals.	[130]

For developing a µBBB platform, the chip should be designed and fabricated in such a way to meet the experimental prerequisites. The sizes of microchannel should be decided according to *in vivo*, microvascular structures, and is achievable with high precision patterning. Secondly, multi-dimensional network structures that create sealed environments in the chip that are independent of one another, and thus look like the *in vivo* microenvironment need to be fabricated. In these models, the cell performance alters significantly from 2D to 3D with additional physiological information and predictive data. Moreover, with the use of microfluidic platforms, it is easy to coalesce and incorporate different functional units within µBBB platforms. Accordingly, the µBBB platforms can be utilized to monitor cell structure, imaging live cells during cell migration experiments, and perform TEER measurement to understand the integrity of µBBB with the help of an inbuilt electrode [21]. Such µBBB platforms present distinctive tools for basic research as well as in drug discovery, neurotoxicity studies, and the development of BBB permeable therapeutic carriers. The key benefit of such tiny µBBB platforms is that the experiments can be performed by using a sub-milliliter quantity of fluids [113].

In µBBB platforms, cell culture media can be continuously passed through the device to provide nutrients to the cells and take-out cellular waste materials. To investigate the cell-cell interactions, the chemical microenvironment around the cells should be precisely controlled and slow enough to monitor during the experiment [114]. Maintaining a relatively similar fluid transport at microscale dimensions is important to achieve similar BBB microenvironment in different experiments [115]. The kinetics of fluid exchange in microfluidic BBB models and cell area can be visualized with a fluorescent dye or dye tagged proteins [116]. Microfluidic channels can be designed to control various flow patterns in such a way to mimic natural blood flow in the microvasculature of BBB [117]. In a dynamic cell seeded microfluidic system like BBB constructs, establishment of a universal flow-pattern-map is not realistic because of the underlying differences in fluid dynamics due to the wettability, roughness of the channel surface, differential distribution of biopolymer coating on channels and difference in cell proliferation [118]. Thus, it is important to investigate the flow dynamics of developed BBB models case by case.

Microfluidic BBB devices provide robust opportunities in regulating mass transport of signaling molecules, active agents, drugs and nutrients for biological studies [119]. Minimizing or tuning mass transport in a BBB model is necessary to provide a physiologically relevant BBB microenvironment. In general, mass transport in microfluidic platforms occurs by Fickian diffusion and convection. Porosity of separation membrane, microchannel dimensions, media flow rate, and flow direction have been found to influence mass transport in microfluidic platforms [120]. In platforms without cell seeding, convection may predominate over diffusion due to the continuous fluid flow through microfluidic channels. However, mass transport across cell seeded microfluidic platforms is also influenced by the extent of transcellular or paracellular transport [121]. Moreover, it is important to confine the mass transport across BBB construct only through the engineered tight junctions mediated by transcellular and paracellular transport. Many of these subjects have already been covered in different original articles [22] or reviews [72,122] by others, so readers may refer them for detailed information.

Currently, many companies manufacture and market readymade microfluidic BBB chips that can be customized for specific applications such as metastases research, drug permeability studies and studying the tight junction integrity. For example, commercially available SynVivo BBB microfluidic chip can be used for the development of such platforms without much facilities and expertise for microfluidic devise development [123,124]. OrganoPlate (Mimetas BV) is another readymade organ-on-a-chip platform that can be used for developing various BBB models suitable for specific applications [125].

### 4.1 Design of bioengineered microfluidic platforms

**Fig. 3:**
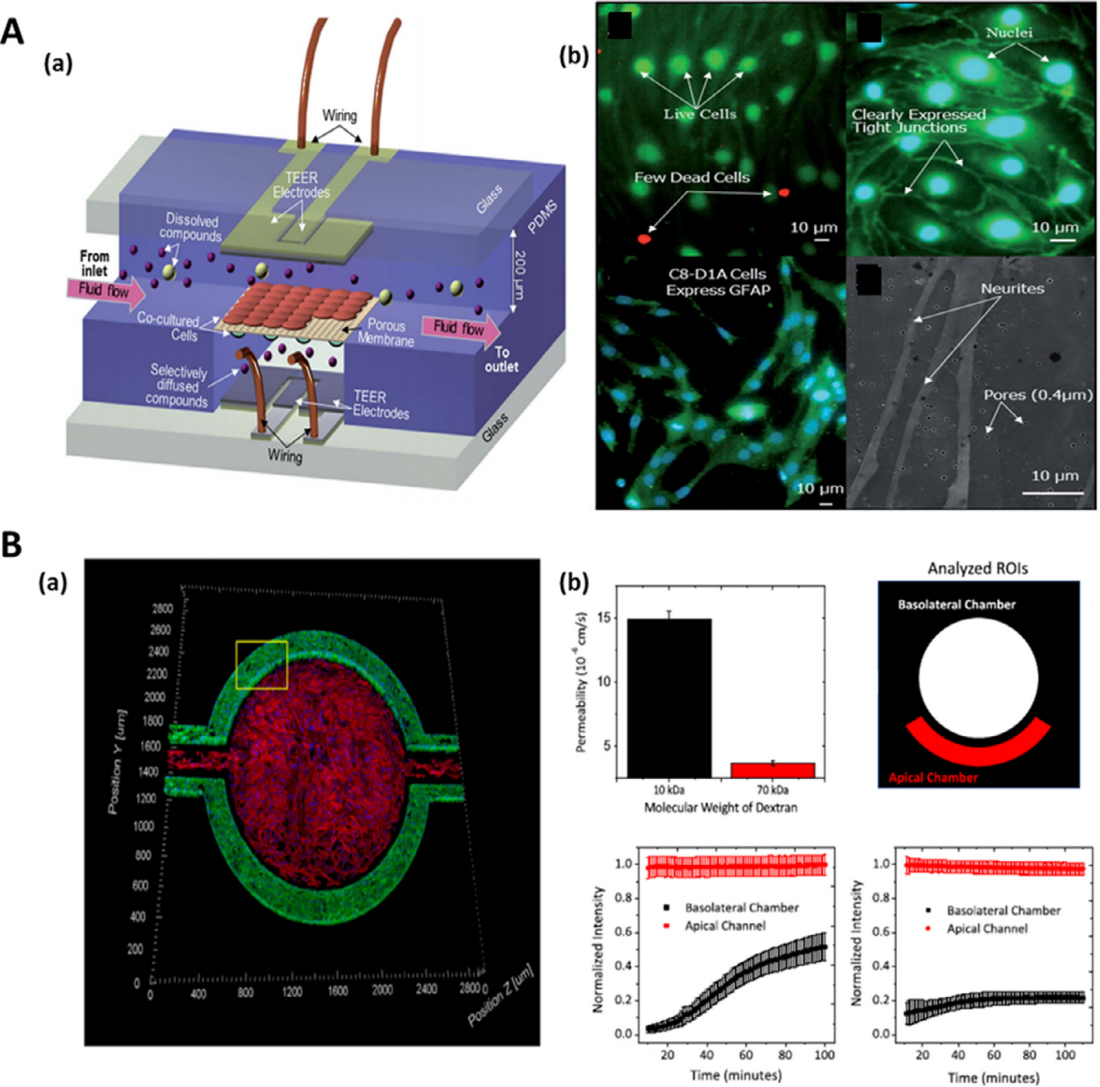
Standard design µBBB platforms **A.** (a) Typical design of the developed µBBB comprising of two perpendicular flow channels. (b) Microscopic images showing the cells seeded in µBBB. Live/Dead stain (green and red colors indicate live and dead cells, respectively) of bEnd.3 cells on day 3 of culture (top left), Immuno-stains of tight junction component ZO-1 (green) (top right), Immunostaining of GFAP (green) in C8-D1A cells indicates astrocytic phenotype on supporting membrane (bottom left), ESEM of C8-D1A neurites on supporting membrane (bottom right). **B.** (a) Co-culture of hCMEC/D3 cell lines (green) and human astrocytes (primary cells) (red) in µHuB. (b) Cellular permeability (Pe) of dextran with various molecular weights through the µBBB platform (top left). Example normalized intensity profiles of transport for a single device with 10 kDa dextran tracer (bottom left) and 70 kDa dextran tracer (bottom right). Fig. A is reproduced from [132] with the permission of Royal Society of Chemistry. Fig. B is reproduced from [124] with Creative Commons Attribution International -0.4 (CC-BY-0.4) license. (For interpretation of the references to color in this figure legend, the reader is referred to the web version of this article.)

Most microfluidic platforms employ porous membrane compartmentalization to produce sandwich assemblies that resemble BBB, vascular channel, and brain component as in the native vasculature-BBB-brain interface. ECs and the other component cells such as pericytes and astrocytes are seeded and allowed to proliferate on each side of the separating membrane. Separation of microvascular endothelial cells from other cells can also be done using trapezoidal structures, micro-gaps, or porous tubular constructs. Fig. 3 shows a typical scheme of the µBBB platform. Choi *et al* fabricated a microfluidic chip for developing bioengineered microenvironment systems consisting of tubular cell structures that mimic BBB [131]. It was composed of a microchip with a tiny chamber loaded with ECM with a few adjoining tubular channels. The ECs seeded and cultured into these channels could stick on to the ECM and form perfusable hollow tissue structures attached to fluid reservoirs with passages for fluid flow in the chip.

Silicon, the first-generation microfluidic device base material, is used with MEMS technology to develop microfluidic structures. On the other hand, the silicon microfabrication process is usually costly, prolonged, and needs unique microfabrication conditions [133]. The majority of microfluidic platforms are working with polydimethylsiloxane (PDMS)-based systems because it is less expensive and simply microfabricated. The organosilicon polymer, PDMS is optically translucent, safe, non-flammable, and permeable towards gas and water [134]. Optical transparency is an experimental necessity, while gas/water permeability plays a crucial role in microfluidic cell culture. PDMS devices are made using a mask via a replica molding process which permits the mass-production of chips from one mold. PDMS and other materials are firmly bound to glass, after simple plasma treatment. Hence it is important to undergo plasma treatment or protein coating on the PDMS surface before cell culture experiment to ensure improved cell adhesion and diminish hydrophobicity [135]. The possibility of leaching out uncross-linked free PDMS monomers into the culture medium, which seriously influences cellular phenotype, cannot be neglected [136]. To overcome these limitations, thermoplastics like cyclic olefin copolymer, styrene polymers, and poly (methyl methacrylate) are used for the fabrication of the microfluidic devices. Yet, it is difficult to develop very complex microstructures in microfluidic BBB platforms using for thermoplastics-based materials alone. The separation of luminal and abluminal layers is made possible by using porous membranes in microfluidic platforms [21]. These membranes act as the medium for co-culturing component cells and enable the estimation of permeability of the BBB constructs. Polyester (PES), polycarbonate (PC), polytetrafluoroethylene (PTFE) and polyethylene terephthalate (PET) are the materials usually used for the preparation of porous membranes [137]. Thickness, pore size, and pore density may influence signal transduction between cells on each sides of the membrane. Commonly used membranes have a thickness of around 10 μm, pore diameter 0.2 or 0.4 μm, and densities around 108/mm^2^ [94]. But the thickness of these artificial membranes is relatively high compared to the in vivo membrane thickness that restricts the interaction between endothelial cells and other co-cultured cells [138].

The integration of nanoelectromechanical systems (NEMS), MEMS, interdigital transducers (IDTs), required microelectronics and conformal antenna makes the µBBB, a smart system suitable for detecting and performing a variety of tasks *in vitro* [139]. Also, MEMS or NEMS based actuators, pumps, and valves can manipulate and control fluids in the range of micro- to pico-liters in tiny channels with micrometer dimensions [140]. Data analysis and interpretation spans from cellular to genomic level using microfluidic platforms and also requires the integration of microelectronic components such as sensors, transducers, actuators, etc. [141]. Advanced micro-molding techniques and micro stereolithography are used for making such sensors, actuators, micro-turbines, and micro-engines. Both self-assembly techniques and robotic manipulation are used for the high-level integration of micro components at nanoscale accuracy in the µBBB platforms. Engineered µBBB platforms use either primary mammalian cells or established cell lines of BMECs, astrocytes, pericytes, and neurons [142].

One of the most widely used cells in µBBB platforms are the BMECs that contribute towards the tight junction integrity of µBBB. Also, cell lines such as hCMEC/D3 (human adult cerebral microvascular endothelial cell line), RBE4 (rat brain endothelial cell line) [143], bEnd.3 (murine brain endothelial cell line) [96], MDCK-MDR1 (multidrug resistance gene MDR1 expressing Mardin-Darby canine kidney cells) [144] are also cultured in µBBB platforms [145]. In contrast with primary cells, immortalized cell lines reduce the workload and decrease the time needed to attain cell confluence. However, some reports showed immortalized cell lines causing leaky barriers and found that it is difficult to form perfect tight junctions [146]. Human cells are more favored over other mammalian cells because of the species level difference at the cellular and molecular level between different organisms. Adriani *et al.* developed BBB structure in a microfluidic chip with human umbilical vein endothelial cells (HUVECs), co-cultured with rat astrocytes and neurons which formed intact monolayer and intercellular junctions in µBBB [147]

Along with microvascular endothelial cells, astroglia cells play a major role as mediators in BBB formation and function. Their presence results in high expression of barrier-relevant proteins, with several studies demonstrating improved BBB physiological parameters including trans-endothelial resistance and decreased paracellular permeability [148]. When ECV304 and rat glioma C6 cells were co-cultured, the resulting monolayers lack the needed rigidity of tight junction and resulted in inferior barrier performance [149]. In another study, BMECs derived from human induced pluripotent stem cells (hiPSCs) were co-cultured with rat primary astrocytes and observed TEER levels peaked between 2000 and about 4000 Ω•cm^2^ [21]. These levels are the highest values observed in the reviewed microfluidic platforms. Due to its potential as a personalized platform, this model holds great promise for person-specific drug penetrability testing. Non-cerebral cells, such as MDCK or Caco-2 are not a good choice for µBBB constructs as these cells differ from microvascular endothelial cells in that they possess less squamous morphology, smaller cell surface area, and different transverse patterns of tight junctions [150]. In an interesting study that evaluated the effect of pericytes on BBB integrity, α-synuclein was added to rat brain endothelial cells (RBECs) - rat brain pericytes co-culture system [151]. Without pericytes, the luminal and abluminal insertion of α-synuclein displayed no alteration of the permeability of the RBEC monolayer. The results suggest that monomeric α-synuclein can compromise the BBB integrity by interacting with pericytes, which in turn suggests the importance of using pericytes in engineered µBBB platforms.

For engineering an appropriate 3D microenvironment, seeding the cells over an ECM component or hydrogel matrix is necessary [117]. Natural biological macromolecules such as fibronectin, collagen, and gelatin were used as a coating material over µBBB platforms [132]. BBB‐EC cultured on brain‐specific EC or multi-cell co-cultures in combination with either pericytes, astrocytes, and glioma cells in the presence of conditioned media derived from these cells can replicate more BBB like properties in µBBB models [152,153].

### 4.2 Characterization of bioengineered µBBB platforms

Characterization of the developed µBBB platforms involves both physicochemical and biological tests. Basic physical tests such as light microscopy and scanning electron microscopy (SEM) are generally performed to understand the microstructural features of the µBBB platforms [154]. Fluid flow test and dye permeability assays are performed to assess the proper fluid mobility through the microchannels. Biological characterization such as cytocompatibility, cell proliferation, and viability is generally performed by culturing BBB specific cells such as microvascular endothelial cells, astroglia cells, pericytes, and neurons over the channels of the µBBB chip [155]. MTT cell viability assay and live/dead staining are generally performed to determine the viability of cells on microfluidic platforms.

**Fig. 4:**
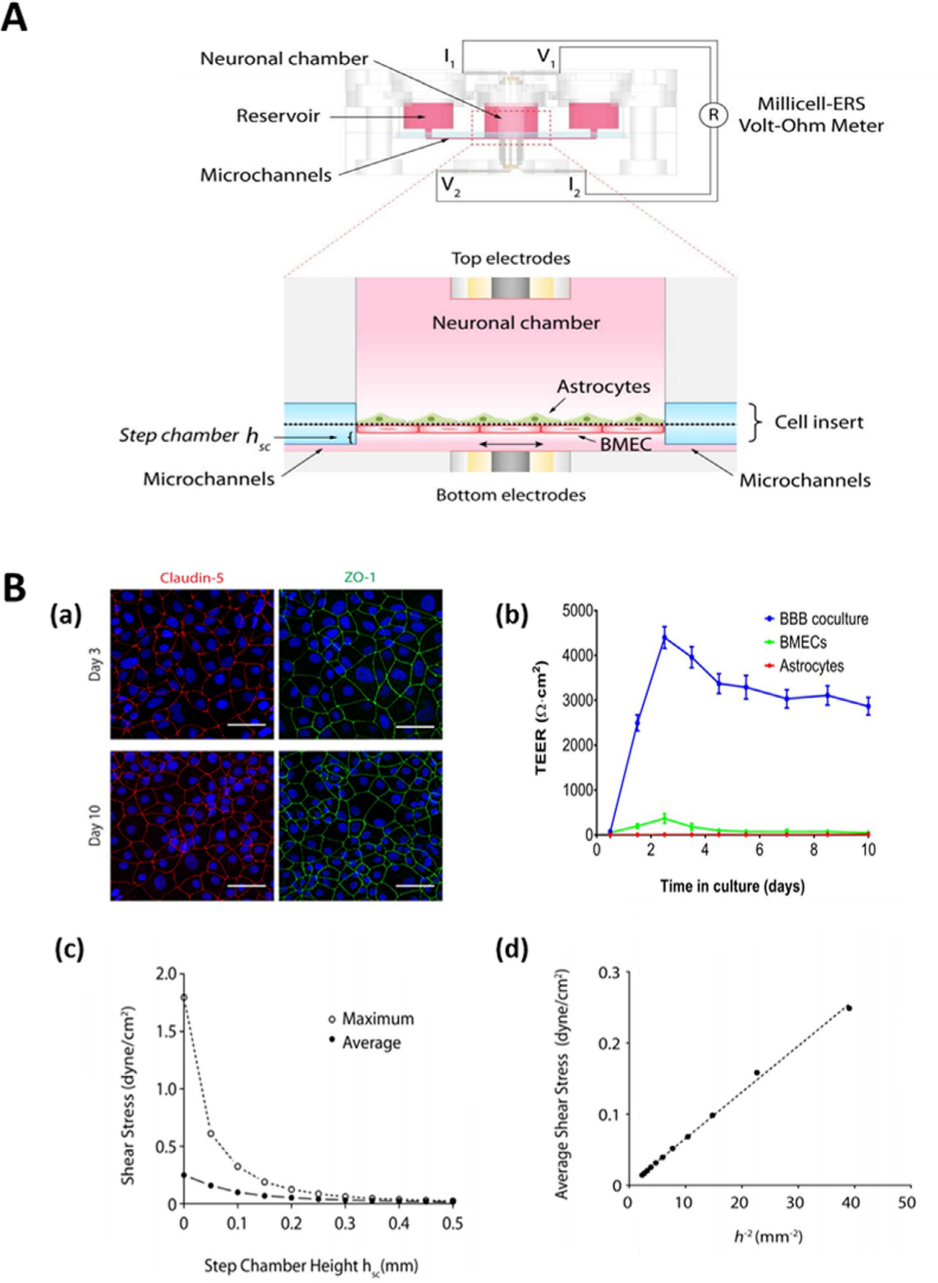
**A.** TEER measurement setup uses a 4-point probe method in which the voltage-sensing electrodes are connected to a Millicell-ERS Volt-Ohm Meter. **B.** (a) The immunostaining images indicated a high-level tight junction protein (claudin-5 and ZO-1) expression showing continuous networks of ZO-1 (green) and claudin-5 (red) outlining the contours of BMECs. (b) The plot of TEER through the culture days obtained from the setup shown above. (c) The simulated plot of the average and maximum shear stresses with respect to step chamber height, (d) simulated average shear stress shows a linear correlation with h^−2^. Reproduced from [21] with the permission of Wiley. (For interpretation of the references to colour in this figure legend, the reader is referred to the web version of this article.)

One of the major properties for all µBBB platforms is that they must greatly limit the paracellular flux of solutes. Typically, those with high trans-endothelial resistance (TEER) and low penetrability of hydrophilic markers and features that may guarantee controlled paracellular and transcellular pathways, are the models of choice. Although the brain endothelium-TEER is greater than 1000 ohm.cm^2^ *in vivo* [95], evidence suggest that reliable data can be gained *in vitro* µBBB platforms if the system shows an adequately high TEER (at least 150 to 200 ohm.cm^2^)[26]. Integration of voltage-sensing instruments and electrodes to the µBBB platforms enable the continuous monitoring of TEER (Fig. 4**A**) [21]. The presence of broad tight junctions holding molecules through inter endothelial cell-cell junctions retains the barrier integrity of BBB [156]. The expression of specific markers can be visualized through immunofluorescence or western blot assays. Visualization of other tight junction proteins such as claudin and ZO-1 by immunofluorescence staining can confirm the formation of tight junctions in µBBB platforms [Fig. 4**B(a**)]. When BMEC and astrocytes were cocultured in the µBBB platform, the TEER value increased from 0-500 (for BMEC and astrocytes monocultures) to 3000-4500. As the penetration of hydrophobic molecules into the µBBB is prevented by a membrane transporter called P-gp efflux pump, the expression of this membrane transporter is also utilized to assess BBB features in microfluidic platforms. As P-gp is important in limiting the entry of several drugs to the CNS, BBB-like P-gp functionality should be assessed particularly in permeability-focused µBBB platforms. Most microvascular endothelial cell-based *in vivo* resemblance co-culture models have not been fully characterized for P-gp functionality with sufficiently large sets of P-gp substrates. Among the models used, the MDCK-multiple drug resistance (MDR)1 model provides the best assessment of P-gp-mediated efflux, although the Caco-2 model also displays a good level of P-gp functionality [157].

Selective transcellular permeability is also a critical characteristic of µBBB platforms, as the passive transcellular pathway is a major route for drugs crossing membrane barriers. *In vivo* brain distribution or *in vivo* permeability data of a reference compound often guides researchers to characterize the reliability of developed µBBB platforms [158]. To evaluate BBB properties in a µBBB model, permeability must be assessed. The relative affinity of a therapeutic agent to the BBB or brain associated cells/tissue can be expressed as the blood-brain partition coefficient [logBB = log (*C*_brain_/*C*_blood_)]. A robust model must have permeability behavior analogous to those found *in vivo*. The selection of an appropriate molecule is important when assessing the permeability [90]. However, sufficient characterization of µBBB platforms requires a chemically diverse set of molecules with a broad range of permeability. A good marker does not affect the structure and property of the formed BBB and should be inert. The model can be further tested by using Evan's blue dye in the permeability assay [159]. In a simple approach, Evan's blue will be added to the microchannels and its propagation to the brain mimicking chamber will be studied over several time points to assess the µBBB permeability. 14C-D-mannitol (182 Da), fluorescein isothiocyanate (FITC) labeled dextran, and 14C-urea (60 Da) are also utilized in permeability studies [160].

Shear stress is generated by the fluid flow in a microfluidic chip, like the flow in normal microenvironments. The shear stress in the cellular channels has a significant influence on endothelial cells in cell growth, structure, cell function, gene expression regulation, and phenotypes [161]. Under shear stress, epithelial cells of diverse origins may express diverse phenotypes [162]. The introduction of fluid flow in a microfluidic chip at physiological levels enhances the key parameters of BBB like increased activity of tight junction proteins improved barrier integrity, which also results in enhanced barrier function. However, significantly decreased levels of fluid shear force encourages the degeneration of tight junction [163]. The shear stress is mostly related to fluid viscosity, flow rates, and geometry of microchannels, flow profile, *etc*. and the stress is 10–20 dynes•cm^−2^ in a 10 μm-diameter capillary in the physiological state [164]. Design and flow channel dimensions significantly influence shear stress. For instance, increasing step chamber height decreases the shear stress values of fluid flow in µBBB platforms [Fig. 4 **B (c, d)]**. A varying flow rate of about 0.01 μL/min to 120 μL/min is observed in microfluidic platforms [165]. Hence, it is imperative to probe and calculate shear stress in a µBBB model by employing simulation software before the fabrication and experimental determination after fabrication.

## 5 Application of bioengineered µBBB platforms in cancer research

Among various *in vitro* models, µBBB platforms are one of the best candidates to apply in BBB research. Several bioengineered µBBB platforms have been designed to use in many research projects. These models are being used to understand the BBB function, to test certain therapeutics, and to evaluate the uptake and delivery of drugs across BBB. One among them with great potential is cancer research. The BBB integrity has a debatable role in brain tumor pathology as well as in drug delivery across BBB and hence, it remains controversial whether the µBBB platforms are an ideal choice of brain tumor research. Studies indicated that disruption of BBB integrity promotes brain metastasis [166]. However, a study showed that a brain penetrating inhibitor, a small molecule designed to cross the intact BBB, can inhibit brain metastases [167]. Such studies using bioengineered µBBB platforms have implications for the future discovery of therapeutic drugs for a better outcome in neuro-oncology patients. They also provide information on developing systemic drugs for preventive strategies for those patients who are susceptible to brain metastasis.

### 5.1 In cancer metastasis research

**Fig. 5:**
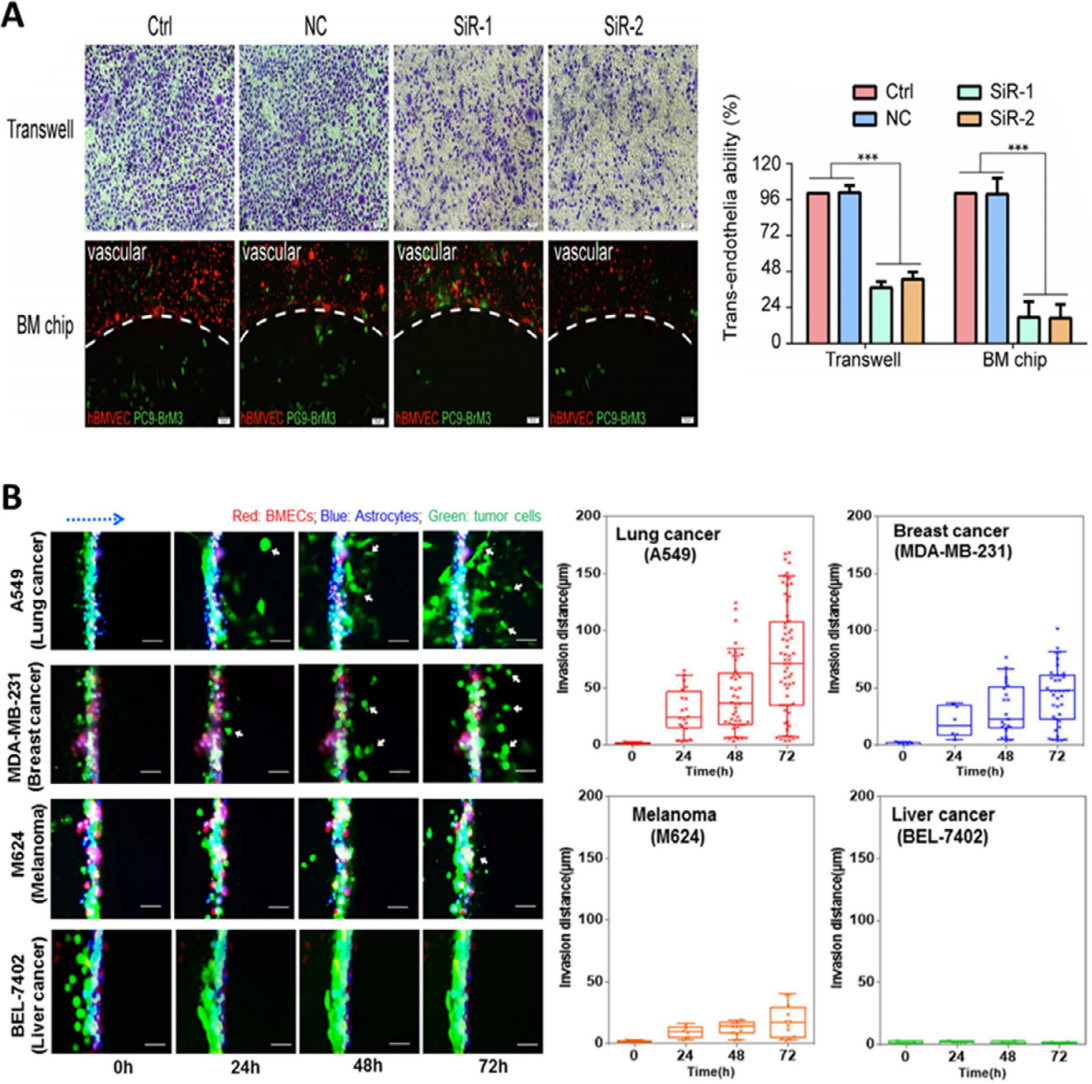
**A.** Representative microscopic images indicating the trans-endothelial cell migration on transwell and cell penetration of the µBBB on the chip **B.** Time-lapse microscopic images of migration of various cancer cells across µBBB system monitored over 72h. Plots of cell migration of various cancer cells crossing the µBBB. Fig. A is reproduced from [169] with the permission of Elsevier. Fig. B is reproduced from [168] with Creative Commons Attribution 4.0 International (CC-BY-4.0) license.

An interesting study characterized a new microfluidic model of the blood tumor barrier (BTB) (with reference to BBB model) that incorporates elements such as flow and induced shear stress on the endothelial cells, utilizing cells such as human umbilical vein endothelial cells co-cultured with CTX-TNA2 rat astrocytes (BBB) or Met-1 metastatic murine breast cancer cells (BTB) [123]. Cells could communicate through microfluidic chambers via a permeable border. This novel microfluidic *in vitro* BTB model can replicate shear stress, permeability, and efflux functions similar to *in vivo* models. In another study, a dynamic 3D microfluidic model was realized to recapitulate the properties of human BBB (Fig. 5). The elements of the system could work together to recapitulate the characteristics of the BBB, which in turn permits the analysis of responses in healthy and diseased microenvironments in the brain [168]. This is attained by utilizing intercellular interactions, cues sensed by mechanoreceptors, and cell motility. This system is claimed to possess the capacity to analyze brain metastasis in human lung, breast, and melanoma cells, and their reactions to chemotherapy. Findings obtained indicate that the communication between the cancer-causing cells and astrocytes might hinder the ability of brain tumor cells to cross into vascular compartments [168]. Non-endothelial NVU cells are critical to the process of provoking BBB phenotypes and normalizing dynamic responses of the BBB to brain activity. Many µBBB models would incorporate these NVU cell types alongside BMECs, providing greater number of options for multiple applications in exploring intricate cellular and fundamental molecular mechanisms of BBB biology.

### 5.2 Cancer drug delivery and drug screening using µBBB platforms

Several types of transport mechanisms play a key role in the transport of different molecules such as nutrients, drugs, and water from blood circulation to brain tissue [170]. The changes in the permeability of the BBB can take place under the influence of several factors including mechanical perturbations or toxic effects of drugs and chemicals. This type of toxins causes contraction of the endothelial cells which in turn causes the damage of the endothelial cells in the BBB [171]. To provide maximum protection to the brain from external harmful agents, BBB filter out almost all the unwanted agents. Hence, it is often hard to reach therapeutically significant dosages of anticancer drugs in the brain [172]. However, drugs and chemicals of high lipid solubility enter the CNS through BBB. Thus, it is most important to design and use µBBB constructs that match the requirements of anticancer drug permeability studies.

Campisi *et al.* developed a µBBB platform to closely mimic the *in vivo* neurovascular organization [173]. Human-induced pluripotent stem cell-derived endothelial cells, brain pericytes, and astrocytes were used in this microfluidic system. The results showed that this 3D-BBB model is an excellent microvascular model serving as a novel and helpful platform for drug screening to envisage neurotherapeutic transport efficiency in pre-clinical applications, especially when compared to the reported static models. A novel three-dimensional neurovascular microfluidic model was developed by Adriani *et al.* combining human cerebral microvascular endothelial cells with primary rat astrocytes and neurons together [147]. Different functional properties and morphological characteristics were exhibited by these three cell types in the neurovascular chip. Size-selective permeability was shown by human cerebral endothelial cells and a quantitative study on neuronal responses was analyzed using the other cell types indicating its applicability for anticancer drug screening. A recent report by Xiaojian *et al.* demonstrated the applicability of a membrane-based µBBB model to study drug delivery into the brain [174]. hCMEC/D3 cells that reconstitute the *in vivo* properties of BBB were cultured on membrane-based microchannels. A model drug, Sunitinib was delivered to the microchannel and tested its permeability across the µBBB model. The advantage of this platform is that it is possible to get the permeability data within 30 min using 5 μL of sample solution by using a quadrupole time-of-flight mass spectrometer (ESI-Q-TOF MS) to quantify the amount of permeated drug.

µBBB platforms that exactly recapitulate the *in vivo* characteristics of BBB for a prolonged period and facilitating the testing of the drug permeability through recirculating perfusion are also inevitable in oncology research. BMECs derived from hiPSCs cultured in a pumpless microfluidic platform along with primary astrocytes derived from the rat constituted such a robust long period testing platform [21]. This model was designed based on the blood residence time of human brain tissues. This allows the screening of drug response during multi-organ interactions. Tae-Eun Park et al. studied the human BBB barrier function using an *in vitro* µBBB model [175]. This model consisting of pluripotent stem cell-derived human BMEC interfaced with primary human brain astrocytes and pericytes. Here in this model, the endothelium possessed elevated levels of tight junction proteins and functional efflux pumps, and it exhibited transcytosis of peptides and antibodies as in *in vivo* conditions. Hence, this model represents a promising platform for testing the permeability of anticancer drugs and therapeutic antibodies transverse the human BBB.

## 6 Challenges and prospects

There are a huge interest and tremendous progress in designing, developing, and testing *in vitro* models that mimic the BBB's distinctive properties in cancer research. Recently, bioengineered µBBB platforms composed of cells, biomaterials, capillary-like microchannels, and electronic components have been developed and utilized in brain metastasis and anticancer drug permeability studies [176]. Such platforms can recapitulate physiological blood circulation as well as shear stress and take up the BBB microenvironment better way compared to other *in vitro* models. Though significant progress has been achieved, bioengineered µBBB models are still in infancy while considering many technological, functional, and commercialization aspects. It can be a challenge to mimic all the properties of *in vivo* animal models in an *in vitro* platform. To use these µBBB platforms as reliable tools, especially in industrial research, many tasks must be accomplished.

The large pore size of membranes used to separate different cell types, limit the direct cell-cell interaction that generally occurs through a matrix. Hydrogels can be used to fill microchannels as an ECM mimetic material to improve cell adhesion and proliferation [177,178], but rigid ECM substrates possess a stiffness higher than those of brain microvessels. Even with the use of ECM mimicking hydrogels, there still would be a remarkable difference between the traits of microfluidic platforms and the associated *in vivo* microenvironment [179]. Some of the crosslinking agents used for hydrogel fabrication can have a deleterious effect on cell viability and proliferation. The diameter of microchannels is a crucial factor that determines the physiological resemblance of bioengineered µBBB platforms with that of natural BBB. However, creating a microchannel with the exact diameter of the capillary is a challenging task [180]. Maintaining the continuous flow of media or seeding cells on the walls of such small channels is not successful so far accompanied by frequent obstruction of the fluid passage. The BBB is highly intricate, unlike available microfluidic platforms that are primarily consisting of only two or three cell types. In co-culture systems, it is challenging to differentiate paracrine and intercellular connections facilitated effects [99]. Moreover, a co-culture model's capabilities are influenced by several attributes, such as cell type and origin, flow rates, and shear stress. These attributes all require optimization for adequate performance levels. The integration of NEMS, MEMS, IDTs, and other required microelectronics in the µBBB platform is required for sensing and controlling its functions. Such precise integration on a microfluidic platform without affecting the function and biological performance is still a challenging task that needs to be addressed with advanced manufacturing and integration techniques. Measurement of various electrical parameters in the aqueous environment of the µBBB device greatly decreases the sensing performance and accuracy of the obtained data.

Almost all the currently available microfluidic devices are prone to handling difficulties associated with several orders of magnitude of difference between conventional laboratory testing kits and microfluidic chips [100]. Several operation details influence model properties and experimental reproducibility. For instance, it is difficult to uniformly seed the cells in a sealed chamber because the upper regions of the channel cannot be seeded by gravity-based cell seeding and due to the presence of air bubbles [181]. Moreover, astrocytes and similar cells are heterogeneous by nature and can therefore be ‘activated’ upon isolation [182], via impact on the BBB‐EC characteristics when used in co‐culture models.

Mitigating issues of experimental reproducibility requires focused detail standardization when it comes to chip fabrication, seeding, and localization [183]. To assess performances or experimental data, standardization of parameter measurement is paramount. As would be expected, current microfluidic models are unable to fully imitate the BBB. Assessing the positives and negatives of microfluidic model designs allows for a selection of an appropriate model along with experimental methods for data adjustment. A long-term objective would be to advance the models as drug screening platforms to evaluate the pathogenesis of neurological diseases. The application of the bioengineered microfluidic model is limited in studying passive paracellular transport and trans-endothelial diffusion, intervened by receptors and transporters. When performing experiments with certain drugs such as chemotherapeutic agents or antiretroviral agents may affect the integrity of the cultured cells. Disturbance in cell-cell binding and damage of tight junction (TJ) proteins significantly affect the barrier function of BBB. Together, it becomes evident that pharmacologic approaches that improve drug penetration of the BBB may possess a positive effect on the treatment responses of brain tumors. The use of some growth factors such as EGF, may induce EMT and result in the changes in cell phenotype and thus tight junctions or barrier properties.

The fluid to endothelial surface area ratio is higher in 2D *in vitro* models than *in vivo* ones. In the absence of other tissues, the fluid‐to‐tissue ratio is enlarged due to scaling down of the volume of the recirculating medium from whole-body blood volume to mimic human blood circulation. Although microfluidic systems can achieve *in vivo*‐like fluid‐to‐tissue volume ratio, the majority of existing models possess a much larger ratio. This is attributed to single‐pass perfusion and complex tubing system. This in turn hinders the effectiveness of recapitulating µBBB as a metabolic source. Future research would benefit from focusing on developing a robust vascular network in 3D bioengineered µBBB models that can improve the fluid to endothelial surface area ratio. In many cases, highly complex designs and sophisticated processes including complex microchannel networks with multiple biomechanical elements are required to effectively simulate the BBB microenvironment *in vitro*. As complexity increases, fabrication becomes more of a challenge. Coincidentally, a large variety of µBBB platforms can be developed via the alteration of minor properties of the structure. Current research uses PDMS as the base chip material, which is not suitable for large-scale device manufacturing and commercialization [80]. Thermoplastics provide a more favorable alternative for industrial purposes, but it is more challenging to achieve complex precise microstructures using them. Until the point of the release of this work, no in vitro BBB model has been accepted as a standard basic model, leaving the choice to be highly dependent on the application and convenience of using the selected model.

## 7 Conclusions

Recent developments of BBB platforms coupled with advanced microfluidic, bioengineering, and imaging technologies have helped the realization of physiologically relevant bioengineered microfluidic BBB models with huge potential in oncology research. Development and use of microfluidic BBB platforms that can recapitulate human BBB both structurally and functionally can overcome several challenges in cancer metastasis and anticancer drug delivery research. Although a lot of progress has already been achieved in the development of bioengineered BBB platforms, there is still a pressing need to address challenges that hinder the large-scale production and commercialization of µBBB platforms. Closer cooperation between academic and industrial community aiming to generate standardized approaches will help to develop platforms that are technically less complicated, cost-effective, and can provide reproducible results.

## Author contributions

Conceptualization: R.A. Investigation: R.A. Resources: R.A. Writing—original draft preparation: R.A., A.H.A., J.K.S., Writing—review and editing: R.A., A.H.A., N.K.S., A.N., A.H. Visualization: S.N.K., R.A. Supervision: R.A., A.H. Project coordination: R.A. Funding acquisition: A.H. All authors have read and agreed to the published version of the manuscript.

## Declaration of Competing Interest

The authors declare that they have no known competing financial interests or personal relationships that could have appeared to influence the work reported in this paper.
